# Variation in the structure of microbial communities associated with different alfalfa (*Medicago sativa* L.) cultivars

**DOI:** 10.3389/fmicb.2025.1676231

**Published:** 2026-01-06

**Authors:** Juan Zhou, Jiahao Li, Shizhuo Dang, Xiaoyan Zhao, Meihua Li, Yingdong Gao

**Affiliations:** 1 Hetao College, Bayannur, China; 2 School of Enology and Horticulture, Ningxia University, Yinchuan, China

**Keywords:** alfalfa cultivars, rhizosphere soil, microbial community, soil factors, association networks

## Abstract

The rhizosphere is a critical zone for root–soil–microbe interactions, and plant species play a major role in shaping its microbial community structure. Alfalfa (*Medicago sativa* L.), a widely cultivated forage crop with high nutritional and economic value, serves as an ideal model for studying cultivar-specific rhizosphere microbiomes. Using high-throughput sequencing, this study aimed to analyze the rhizosphere microbial communities of seven alfalfa cultivars. A total of 27,878 bacterial OTUs and 5,380 fungal OTUs were identified across all samples. The dominant microbial phyla included Actinobacteria and Proteobacteria (bacteria) as well as Ascomycota (fungi). Although the overall microbial community composition was broadly similar across cultivars, some subtle differences were observed. Key bacterial genera such as *Bacillus*, *Arthrobacter*, *Nocardioides*, and *Gaiella* were abundant in the rhizosphere. Notably, the cultivars ‘JN7’ and ‘QJ’ exhibited higher overall microbial abundance than the other cultivars. Soil properties were found to differentially influence microbial abundance across the cultivars. Specifically, soil organic matter, total nitrogen (TN), and total phosphorus (TP) significantly affected bacterial abundance, whereas TP, TN, and available phosphorus were the key drivers of fungal community variation. Network analysis revealed stronger co-occurrence relationships among bacterial taxa than among fungal taxa, and predominantly positive associations were detected. This study provides insights into alfalfa cultivar-associated rhizosphere microbiomes and lays a foundation for the development of targeted biofertilizers and optimized cultivation strategies.

## 1 Introduction

Soils serve as major reservoirs of microbial populations, playing a crucial role in shaping their abundance and diversity (Roesch, 2007). The rhizosphere, often referred to as the plant’s “second genome,” represents a hotspot for root–soil–microbe interactions and harbors the highest number of microorganisms (Zhang et al., 2017). This highly complex and dynamic zone is shaped by plant roots and their associated microbial communities, forming a multifaceted “melting pot” of biological components and processes (Xiong et al., 2021; Singh et al., 2022). Growing evidence highlights the central role of rhizosphere microbiomes in sustaining ecosystem functioning (Lu and Zhang, 2006). Notably, studies have demonstrated that these root-associated microorganisms profoundly influence a range of plant processes, including growth regulation, root architecture optimization, nutrient acquisition and cycling, abiotic stress tolerance, and pathogen protection (Hiruma et al., 2016; Pieterse et al., 2016). Consequently, the microbial diversity and community structure of the rhizosphere are considered important indicators of soil quality and plant health (Kroll et al., 2017).

Several studies have demonstrated that both plant species and genotype are major determinants of rhizosphere microbiome assembly and organization (Sun et al., 2013). Different cultivars can recruit different microbial consortia through root–microbe interactions, potentially altering functional dynamics within the rhizosphere ecosystem (Bais et al., 2006). Plants influence the biodiversity of soil microbes in soils through the release of exudates containing compounds such as amino acids, sugars, organic acids, and signaling compounds that stimulate microbial growth and activity (Dunfield and Germida, 2001; Mommer et al., 2016). In turn, microbes also produce elicitor molecules that trigger metabolic changes in host plants, inducing responses such as the accumulation of secondary metabolites (Chamkhi et al., 2021). Previous studies on *Paeonia lactiflora* Pall (Zu et al., 2022), mulberry (Ou et al., 2019), ginseng (Wang H. et al., 2020), maize (Kong et al., 2020), tomato (Gaete et al., 2021), and rabbiteye blueberry (Jiang et al., 2017) have consistently shown significant cultivar-driven differences in rhizosphere microbial diversity and community structure. This raises a key research question: Do different crop cultivars assemble distinct rhizosphere microbial communities? Addressing this question is crucial for understanding how plant genotypes influence soil microbial communities and for optimizing crop management strategies.

Alfalfa (*Medicago sativa* L.) is a perennial leguminous forage crop extensively cultivated for hay, pasture, and silage production. It is globally recognized for its high economic value and superior forage quality, which are attributed to its high yield and exceptional nutritional content. With an extensive cultivation history, alfalfa has one of the largest planting areas among forage crops worldwide (Li et al., 2020; Yang et al., 2021). Additionally, alfalfa is a potential “microbial hotspot crop” (Samaddar et al., 2021). Recent research on alfalfa and soil microorganisms has mainly focused on factors such as cropping practices, planting years, crop rotation, intercropping, soil fertility, enzyme activity, fermentation-associated microbial communities, soil microbial communities, and alfalfa yield (Beauregard et al., 2009; Li and Wu, 2018; Bai et al., 2020; Samaddar et al., 2021; Zhou et al., 2022). However, studies specifically examining rhizosphere microbial communities across cultivars of plants such as alfalfa remain limited. Therefore, this study employed high-throughput sequencing to characterize both bacterial and fungal communities in the rhizosphere of seven alfalfa cultivars. The objectives were to elucidate the microbial composition and community structure within the rhizospheres of these cultivars, to identify core microbiomes associated with each cultivar, and to investigate the microbial interactions among different alfalfa cultivars. By analyzing the co-occurrence network characteristics of rhizosphere bacterial and fungal communities across different alfalfa cultivars, this study provides a foundation for identifying beneficial microbial taxa with potential applications in promoting plant growth and enabling biological control.

## 2 Materials and methods

### 2.1 Plant material and sample collection

Experiments were conducted in Yinchuan City, Ningxia Province, China (38°48′N, 106°18′E). This region has a mid-temperature arid continental climate, with an annual temperature of 15 °C, annual precipitation of 129 mm, and light loam soil (specifically, light gray calcareous soil). The approximate soil composition is as follows: 58.39% sand particles, 15.86% silt particles, and 25.76% clay particles. For this study, seven cultivars of alfalfa (*Medicago sativa* L.) were selected and grown in the natural environment of this region. Specifically, the cultivars ‘Zhong Mu No.3’(ZM3), ‘Gan Nong No.4’(GN4), ‘Zhong Lan No.2’(ZL2), ‘DF310’(DF), ‘Qi Ji’(QJ), ‘Ju Neng No.7’(JN7), and ‘Algonquin’(AL) were cultivated in a 3 m × 9 m experimental plot under uniform agronomic management (planted on April 19, 2022). Manual seeding was conducted at a depth of 2 cm, with a seeding rate of 22.5 kg/hm^2^ and a row pitch of 15 cm. The cultivation site, previously used to grow corn, underwent standardized tillage and soil homogenization before planting to ensure edaphic consistency across all cultivars. Typically, four cuts of these plants are harvested per year. However, in the first planting year, it is common to harvest only three cuts.

For this study, the plants were harvested at the optimal time point, i.e., the early flowering stage (10% flowering) (August 5, 2022). Random soil samples were collected from the agricultural field, and rhizosphere soil samples were obtained from every cultivar through standardized collection methods. The rhizosphere soil samples were collected from individual plants using a clean spade at a depth of 0–20 cm. The loosely adhered soil aggregates were removed, and the rhizosphere soil attached to the roots was then collected using sterile brushes (Zhang et al., 2023). The samples were immediately frozen in liquid nitrogen and stored at −80 °C before microbial analysis. Meanwhile, parallel bulk soil samples were air-dried for physicochemical characterization.

### 2.2 Determination of soil physicochemical properties

Soil available nitrogen (AN) was quantified via alkaline hydrolysis diffusion, while soil total nitrogen (TN) was assessed using micro-Kjeldahl digestion with H_2_SO_4_ followed by steam distillation. Total phosphorus (TP) was digested using H_2_SO_4_–HClO_4_, and available phosphorus (AP) and available potassium (AK) were extracted with sodium bicarbonate and ammonium acetate, respectively, followed by determination using a molybdenum-blue spectrophotometer (Fu et al., 2021). Soil organic matter (SOM) content was measured by wet digestion with the potassium dichromate method. Soil pH was determined with a pH meter (Leici PHSJ-4F, Shanghai Yidian Scientific Instrument Co., Ltd., Shanghai, China) using a soil/water suspension (1:5 w/v). Electrical conductivity (EC) was determined with a conductivity meter (Leici DDSJ-318, Shanghai Yidian Scientific Instrument Co., Ltd., Shanghai, China) using the 1:5 soil/water extraction method (Liu et al., 2022).

### 2.3 DNA extraction and PCR amplification

Total microbial DNA was isolated from the rhizospheres of different alfalfa cultivars using the FastDNA® SPIN Kit for Soil (MP Biomedicals, Southern California, USA). DNA integrity was verified using 1% agarose gel electrophoresis, and DNA purity and concentration were determined using a NanoDrop 2000 spectrophotometer (Thermo Scientific, Wilmington, USA). Based on previous reports, the primer pairs ITS1F (5’-CTTGGTCATTTA GAGGAAGTAA-3′) and ITS2R (5’-GCTGCGTTCTTCATCGAT GC-3′) (Ju et al., 2022), and 338F (5’-ACTC CTAC GGGA GGCA GCAG −3′) and 806 R (5’-GGAC TACH VGGG TWTC TAAT −3′) (Liu et al., 2015) were used to amplify the fungal internal transcribed spacer (ITS) region and bacterial 16S rRNA gene, respectively. PCR amplification was performed using an ABI GeneAmp® 9,700 PCR thermocycler (ABI, CA, USA). Each 20 μL reaction mixture (including the water control) contained 10 ng template DNA, 4 μL 5 × FastPfu buffer, 2 μL 2.5 mM dNTPs, 0.8 μL of each primer (5 μM), 0.4 μL TransStart® FastPfu DNA Polymerase, and ddH_2_O. The PCR steps were as follows: initial denaturation at 95 °C for 3 min; 27 cycles of denaturation at 95 °C for 30 s, annealing at 55 °C for 30 s, and extension at 72 °C for 45 s; followed by a final extension at 72 °C for 10 min. The PCR products were checked on a 2% (w/v) agarose gel, purified using the AxyPrep DNA Gel Extraction Kit (Axygen Biosciences, Union City, CA, USA), and quantified using a Quantus™ Fluorometer (Promega, USA).

### 2.4 Illumina MiSeq sequencing and data processing

Purified amplicons were sequenced on the Illumina MiSeq PE300 platform (Illumina, San Diego, USA) by Majorbio Bio-Pharm Technology Co., Ltd. (Shanghai, China) according to standard protocols. Paired-end reads were quality-filtered using fastp version 0.19.6^1^ to remove ambiguous bases (Chen et al., 2018), and read merging and quality trimming (Q < 20) were performed using FLASH (version 1.2.11) (Magoč and Salzberg, 2011). Adapter/primer sequences and short reads (<10 bp), as well as chimeric sequences, were eliminated using QIIME version 1.9.1 (Dorjgochoo et al., 2023). The operational taxonomic units (OTUs) were clustered at a 97% similarity threshold using the UPARSE software (version 11) (Edgar, 2013). Bacterial OTUs were taxonomically annotated using the RDP Classifier (version 2.2) with the SILVA database (version 138; 16S/18S rDNA), while fungal sequences were annotated using the BLAST algorithm against the UNITE ITS database (version 8.0) (De Corato et al., 2020). The PICRUSt (Douglas et al., 2020) and FUNGuild (Schmidt et al., 2018) databases were used to predict the function of the identified bacteria and fungi, respectively.

### 2.5 Statistical analysis

One-way analysis of variance was used to test for differences in soil chemical properties among the different treatment groups. Tukey’s Honest Significant Difference (HSD) test was applied for multiple comparisons. Using the Majorbio Cloud platform^2^, we conducted OTU-based bioinformatic processing. Mothur version 1.30.1 was used to calculate rarefaction curves and alpha diversity indices (observed OTUs, Chao1 richness, Shannon index, PD index, and Good’s coverage). Microbial community composition was assessed using PCoA (based on Bray–Curtis distance) with the vegan v2.5–3 package (Mei et al., 2022). Furthermore, PERMANOVA based on Bray–Curtis distance was used to quantify cultivar-induced variations in bacterial and fungal assemblages. Variance inflation factors (VIFs) for soil variables were calculated using the car package VIF function (version 2.5–3), and distance-based redundancy analysis (db-RDA) was performed with the vegan v2.5–3 package to elucidate soil–microbe relationships. Microbial co-occurrence networks were constructed using Spearman’s correlation coefficients (|*ρ*| > 0.5, *p* < 0.05, Benjamini–Hochberg corrected) for dominant bacterial and fungal genera with an average relative abundance >0.1%. Network topological properties (e.g., average path length, modularity) were computed using R packages such as igraph to reveal non-random assembly patterns in microbial communities, and visualization was performed using Gephi (Barberán et al., 2012).

## 3 Results

### 3.1 Sequencing, rhizosphere microbial diversity, and abundance

Using Illumina sequencing, the impact of plant cultivars on rhizosphere microbial communities (bacteria and fungi) was assessed. After filtering chimeric sequences and mismatches, a total of 1,307,126 and 1,460,458 high-quality sequences were obtained for bacteria and fungi, respectively, from all 21 rhizosphere soil samples. The number of valid tags per sample ranged from 60,038 to 75,516. For bacteria, the cultivar with the highest number of sequences was ZM3 (65,169 sequences), whereas GN4 had the lowest number of bacterial sequences (59,598 sequences). For fungi, JN7 contained the greatest number of sequences (75,516), while GN4 had the lowest number (65,112).

Cluster analysis at a 97% similarity threshold yielded 27,878 bacterial OTUs and 5,380 fungal OTUs in the rhizosphere soil of the various alfalfa cultivars (Table 1). The bacterial OTU richness (*n* = 3,983) was higher than the fungal richness (*n* = 769) across the seven cultivars. Additionally, the number of bacterial OTUs in ZM3 (*n* = 4,837) was higher than that in JN7 (*n* = 4,391). The rarefaction curves for the 21 samples approached saturation (Supplementary Figure S1), demonstrating that the sequencing depth was sufficient to comprehensively represent the target microbial communities.

**Table 1:** Diversity indexes of microbial communities associated with alfalfa rhizosphere soil.

Microbial community	Similarity level of 97%							
	Samples	Valid tag	OTU	Simpson	Shannon	Ace	PD	Coverage/%
Bacteria	ZM3	65,196	4,128	0.996 ± 0.00a	6.63 ± 0.03a	3,966 ± 56.90a	253.94 ± 2.70a	97.01a
	GN4	59,598	4,094	0.995 ± 0.00a	6.54 ± 0.20abc	3748.45 ± 307.32ab	243.11 ± 17.96ab	97.19ab
	ZL2	60,125	3,939	0.996 ± 0.00a	6.35 ± 0.15c	3706.74 ± 179.85ab	238.19 ± 12.35abc	97.20ab
	DF	64,524	4,075	0.995 ± 0.00a	6.62 ± 0.09ab	3840.06 ± 82.12ab	245.43 ± 6.30ab	97.11ab
	QJ	60,038	3,987	0.995 ± 0.00a	6.51 ± 0.08abc	3603.49 ± 244.66ab	229.36 ± 6.76bc	97.30ab
	AL	64,269	4,017	0.994 ± 0.00a	6.55 ± 0.10abc	3864.84 ± 132.55ab	243.47 ± 10.03ab	97.09ab
	JN7	61,960	3,638	0.995 ± 0.00a	6.36 ± 0.17bc	3491.60 ± 102.9b	220.17 ± 7.02c	97.37a
Fungi	ZM3	67,465	709	0.86 ± 0.12a	3.12 ± 0.66a	626.47 ± 73.34ab	97.73 ± 13.73a	99.80ab
	GN4	65,112	797	0.92 ± 0.02a	3.56 ± 0.09a	620.16 ± 48.36ab	108.3 ± 13.28a	99.79ab
	ZL2	66,497	763	0.90 ± 0.03a	3.40 ± 0.14a	627.21 ± 49.69ab	111.2 ± 15.55a	99.78ab
	DF	70,626	777	0.91 ± 0.02a	3.38 ± 0.17a	590.09 ± 64.99ab	108.93 ± 5.56a	99.83ab
	QJ	67,327	757	0.92 ± 0.01a	3.51 ± 0.38a	541.38 ± 24.16b	101.75 ± 10.77a	99.83a
	AL	74,275	824	0.94 ± 0.01a	3.64 ± 0.33a	724.03 ± 91.30a	118.06 ± 18.13a	99.79b
	JN7	75,516	753	0.91 ± 0.02a	3.39 ± 0.46a	595.43 ± 65.54ab	104.85 ± 16.89a	99.83ab

Significant differences were detected through comparisons between different cultivars at the same site; Data are shown as the mean ± standard deviation (*n* = 3). Different lowercase letters (a, b, c) indicate a significant difference at *p* ≤ 0.05.

**Figure 1:**
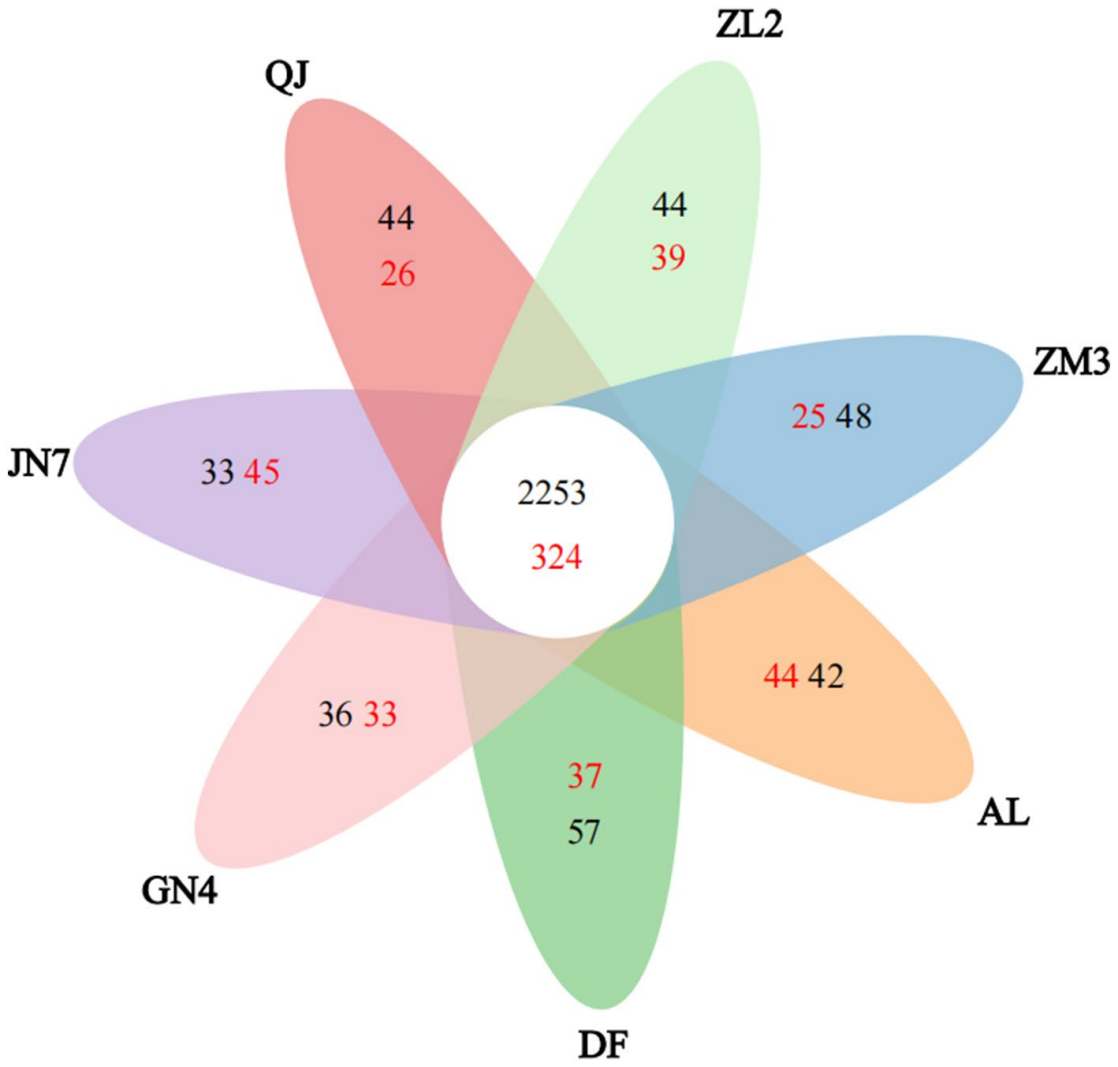
Venn diagrams showing shared bacterial (black) and fungal (red) OTUs across the rhizospheres of the seven alfalfa cultivars.

The number of OTUs differed among the seven alfalfa cultivars. The Venn diagram in Figure 1 shows the shared and unique bacterial OTUs among the different cultivars. The shared OTUs, representing the core microbiome, included 2,253 bacterial and 324 fungal OTUs, respectively. Moreover, the number of unique OTUs ranged from 33 (JN7) to 57 (DF) for bacteria and from 25 (ZM3) to 45 (JN7) for fungi. These data demonstrated the distinct rhizosphere microbial community profiles of different alfalfa cultivars, with each cultivar exhibiting specific bacterial and fungal compositions, diversity patterns, and proportions of unique microorganisms.

Microbial diversity analysis of the seven alfalfa cultivars was conducted based on multiple alpha-diversity indices: community diversity (Simpson), community complexity (Shannon), taxonomic richness (Ace), sequence depth (coverage), and phylogenetic breadth (PD) (Table 1). The coverage value, an indicator of sequencing depth adequacy, exceeded 97% for bacteria and 99% for fungi across all samples, confirming that the data covered the majority of rhizosphere species. Alpha-diversity assessment revealed significant variations in bacterial community diversity, evenness, and richness among the different alfalfa cultivars, whereas richness was the only fungal index showing significant differences (Table 1). Among the cultivars, ZM3 exhibited the highest bacterial diversity values, including the highest Shannon index, Ace richness, and PD (Ace: 3966 ± 56.90; Shannon: 6.63 ± 0.03; PD: 253.94 ± 2.70), while JN7 showed the lowest values (Ace: 3491.60 ± 102.9; Shannon: 6.36 ± 0.17; PD: 220.17 ± 7.02). For fungi, the highest richness was detected in AL (Ace: 735.32 ± 97.67; Shannon: 3.64 ± 0.33), whereas cultivar QJ presented the lowest richness (Ace: 541.383 ± 24.16; Shannon: 3.51 ± 0.38). Overall, both bacterial and fungal communities in the rhizosphere soil of the cultivars ZM3 and AL exhibited higher species richness.

### 3.2 Microbial community composition

**Figure 2:**
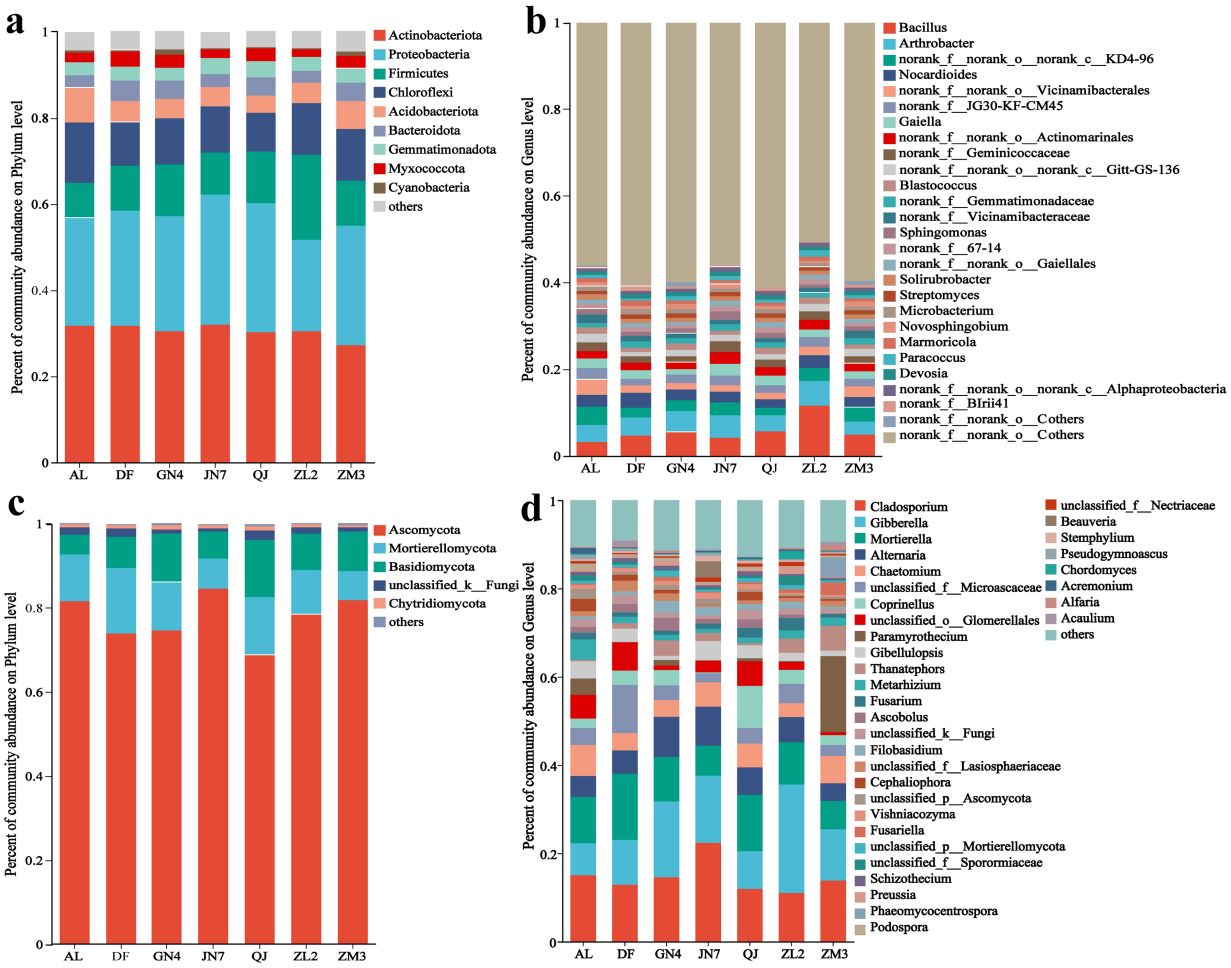
Relative abundance of different bacterial phyla **(a)**, bacterial genera **(b)**, fungal phyla **(c)**, and fungal genera **(d)**.

The bacterial OTUs in the rhizosphere were annotated to 1 kingdom, 36 phyla, 125 classes, 305 orders, 505 families, 982 genera, and 2,039 species, while the rhizosphere fungal OTUs were annotated to 11 phyla, 36 classes, 70 orders, 158 families, 308 genera, and 545 species. Overall, the bacteria were more abundant than the fungi in terms of composition and distribution. Samples from the rhizosphere soils of different cultivars were analyzed for microbial community composition (Figures 2a, c). Among bacterial phyla (Figure 2a), Actinobacteria demonstrated the highest relative abundance (27.59–31.86%), followed by Proteobacteria (21.26–30.19%), Firmicutes (8.11–19.62%), and Chloroflexi (8.94–14.07%), with these phyla collectively representing >80% of all sequences. Notably, Proteobacteria peaked in JN7 (31.09%) and showed the lowest relative abundance in AL (24.94%), DF (26.48%), and ZL2 (21.26%). Compared with other cultivars, QJ exhibited the most diverse bacterial composition at the phylum level. Meanwhile, fungal communities were overwhelmingly dominated by Ascomycota (68.84–84.61%), while Mortierellomycota (6.82–15.71%) accounted for 82.55–92.77% of the total. Ascomycota abundance was particularly pronounced in JN7 (84.61%), ZM3 (81.94%), and AL (81.61%) (Figure 2c). Additionally, fungal profiles displayed remarkable consistency across cultivars despite their genetic differences.

The top 30 bacterial and fungal genera by relative abundance were analyzed further (Figures 2b, d). Among bacterial genera, *Bacillus* showed the highest prevalence (3.22–11.6%), followed by *Arthrobacter* (3.11–5.75%), *Nocardioides* (1.92–3.38%), and *Gaiella* (1.73–2.64%) (Figure 2b). *Bacillus* was the dominant bacterial genus in ZL2, QJ, GN4, ZM3, DF, JN7, and AL, with relative abundances of 11.66, 5.72, 5.56, 4.92, 4.77, 4.37, and 3.22%, respectively. The most abundant fungal genus was *Cladosporium* (11.03–22.38%), with abundances of 11.03, 11.97, 12.88, 13.85, 14.59, 15.56, and 22.38% in the rhizosphere soils of ZL2, QJ, DF, ZM3, GN4, AL, and JN7, respectively. This was followed by *Gibberella* (7.24–24.59%), *Mortierella* (6.40–14.99%), *Alternaria* (4.04–9.10%), and *Chaetomium* (3.13–7.03%) (Figure 2d). Distinct fungal profiles were observed among the different cultivars, with JN7 dominated by *Cladosporium* (22.38%), DF by *Mortierella* (14.99%), ZL2 by *Gibberella* (24.59%), GN4 by *Alternaria* (9.10%), AL by *Chaetomium* (7.03%), and ZM3 by *Paramyrothecium* (17.20%). Several fungal taxa, such as *Gibellulopsis*, *Thanatephorus*, *Metarhizium*, *Fusarium*, and unclassified_*Glomerellales*, also varied in composition among cultivars. Overall, different alfalfa cultivars shared the same dominant species at the phylum and genus levels, but the abundances of these microbes differed.

Using LEfSe differential analysis, seven significant biomarkers were identified in the rhizosphere soils of the alfalfa cultivars (LDA score > 2). Among these, *Bacillus* (LDA score > 4.7) contributed most significantly to the differences among the cultivars (Supplementary Figure S3). This was followed by the bacterial genera *Bacteroidetes* (LDA score > 4.0), *Gammaproteobacteria* (LDA score > 4.4), and *Polyangia* (LDA score > 3.8) (Supplementary Figure S3), as well as the fungal genera *Acaulium* (LDA score > 3.6), *Pyxidiophora* (LDA score > 3.1), and *Gymnoascus* (LDA score > 2.7) (Supplementary Figure S2). These findings indicated that different plant cultivars may construct their rhizosphere microenvironments by selecting distinct microbial communities.

### 3.3 Microbial functional groups in different cultivars

**Figure 3:**
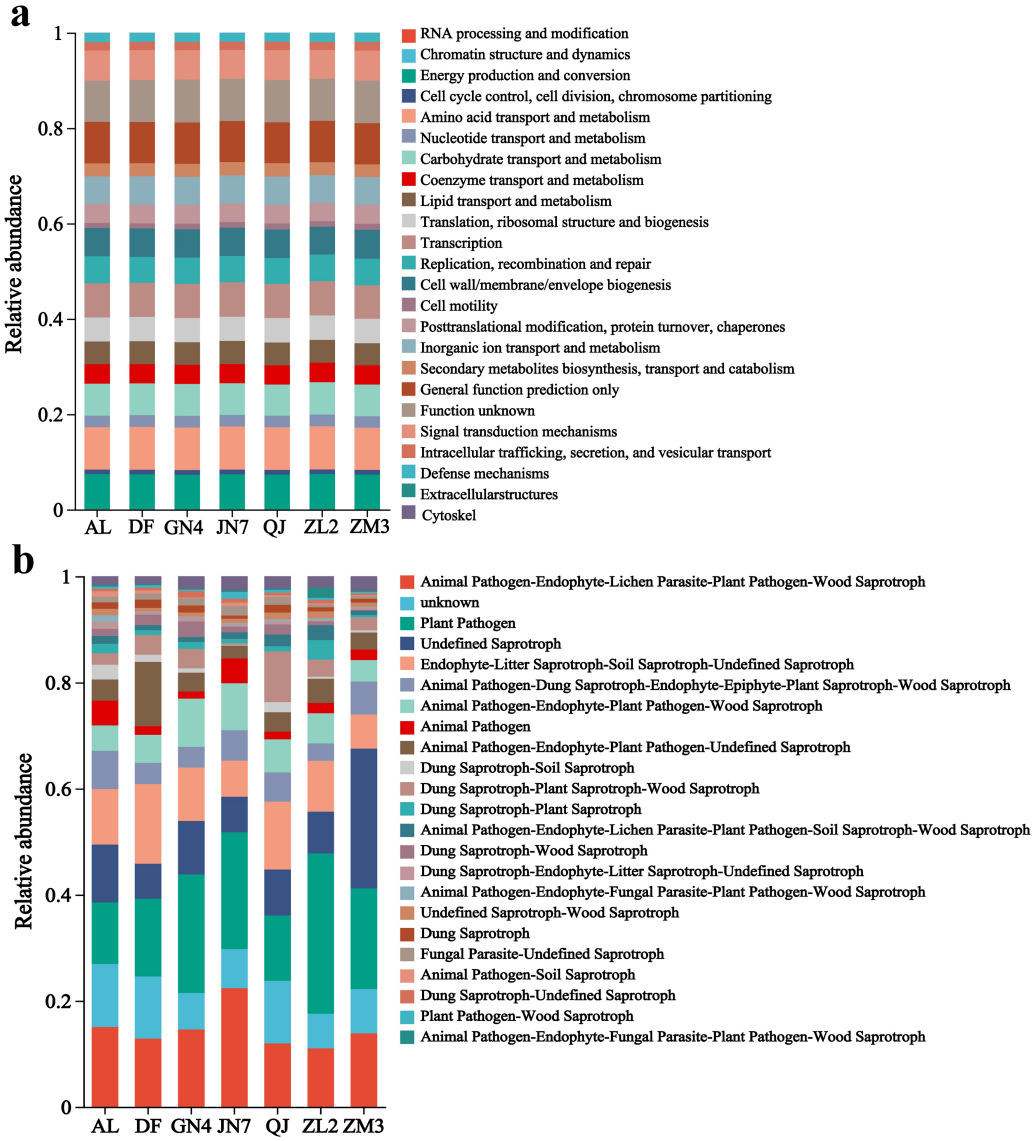
Bacterial **(a)** and fungal **(b)** functional groups in the rhizosphere soil of different cultivars.

PICRUSt analysis predicted that the bacterial communities in the rhizospheres of alfalfa were involved in 24 major metabolic functions represented in the KEGG database. Across all samples, the functional groups related to amino acid metabolism (AAM) and energy production and conversion (EPC) were the most abundant, followed by those associated with transcription, carbohydrate transport and metabolism (CTM), signal transduction mechanisms (STM), and cell wall/membrane/envelope biogenesis. Functional groups involved in inorganic ion transport and metabolism (ITM), replication, recombination, and repair (RRR), and lipid transport and metabolism (LTM) were also significantly more prominent than other functional groups. The relative abundance of AAM-associated bacteria in the cultivars AL, DF, GN4, JN7, QJ, ZL2, and ZM3 was 8.88, 8.98, 8.93, 9.05, 8.94, 8.05, and 8.85%, respectively. Meanwhile, EPC-related bacteria showed relative abundances of 7.37, 7.29, 7.20, 7.31, 7.23, 7.34, and 7.25% in these cultivars; transcription-associated bacteria, 7.17, 7.13, 7.15, 7.23, 7.18, 7.19, and 7.03%; CTM-related bacteria, 6.72, 6.65, 6.69, 6.64, 6.53, 6.77, and 6.63%; and STM-related bacteria, 5.76, 5.85, 5.89, 5.9, 5.91, 5.81, and 5.85% (Figure 3a).

FUNGuild analysis categorized fungal trophic modes into symbiotroph, saprotroph, and pathotroph groups, identifying nine nutritional types and 49 functional groups, of which 24 major groups were analyzed (Figure 3b). Although the functional categories were broadly similar among samples, their relative abundances differed markedly. Groups associated with Animal Pathogen–Endophyte–Lichen Parasite–Plant Pathogen–Wood Saprotroph (11.03–22.38%), Plant Pathogen (11.63–30.21%), and Undefined Saprotroph (6.60–26.33%) were the most abundant. Pathogenic fungi were predominantly represented by plant and animal pathogens, showing significantly higher abundance levels than other functional groups.

### 3.4 Inter-species differences in microbial community structure

**Figure 4:**
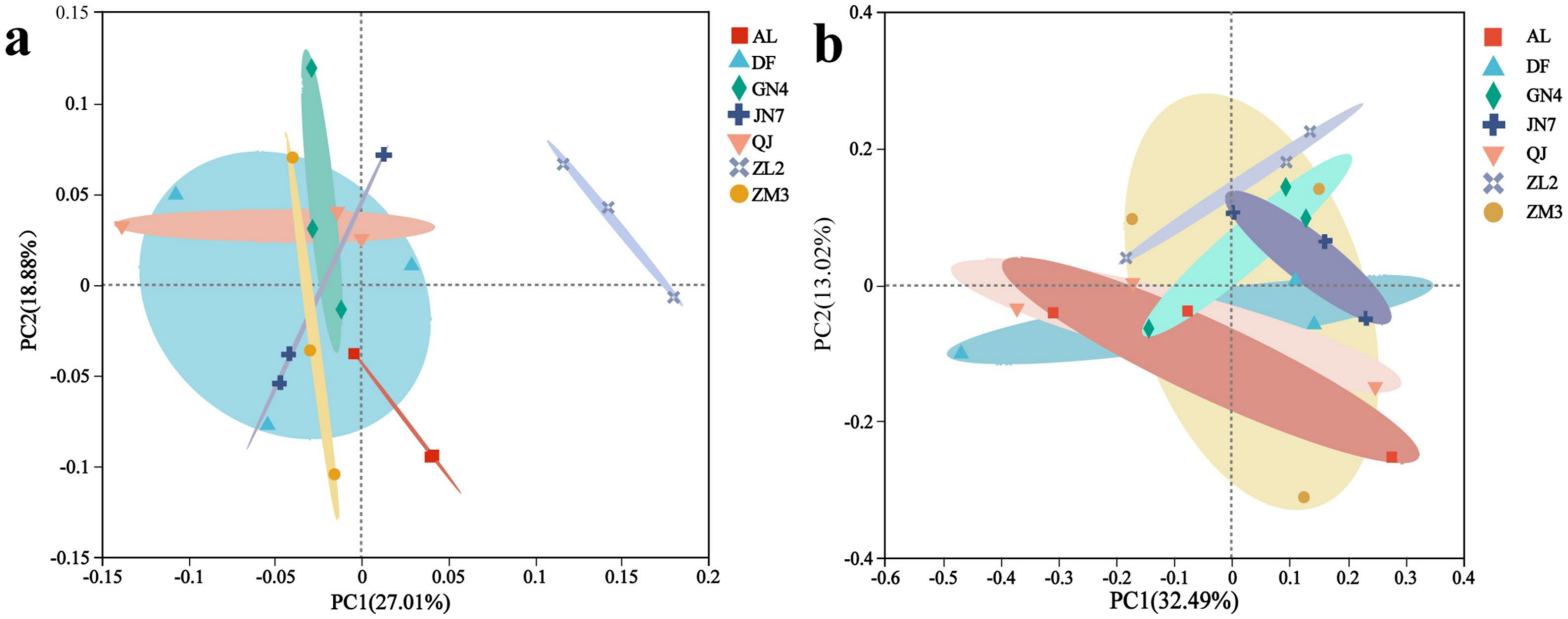
Principal coordinate analysis of microbes in the seven alfalfa cultivars: **(a)** bacteria; **(b)** fungi.

**Figure 5:**
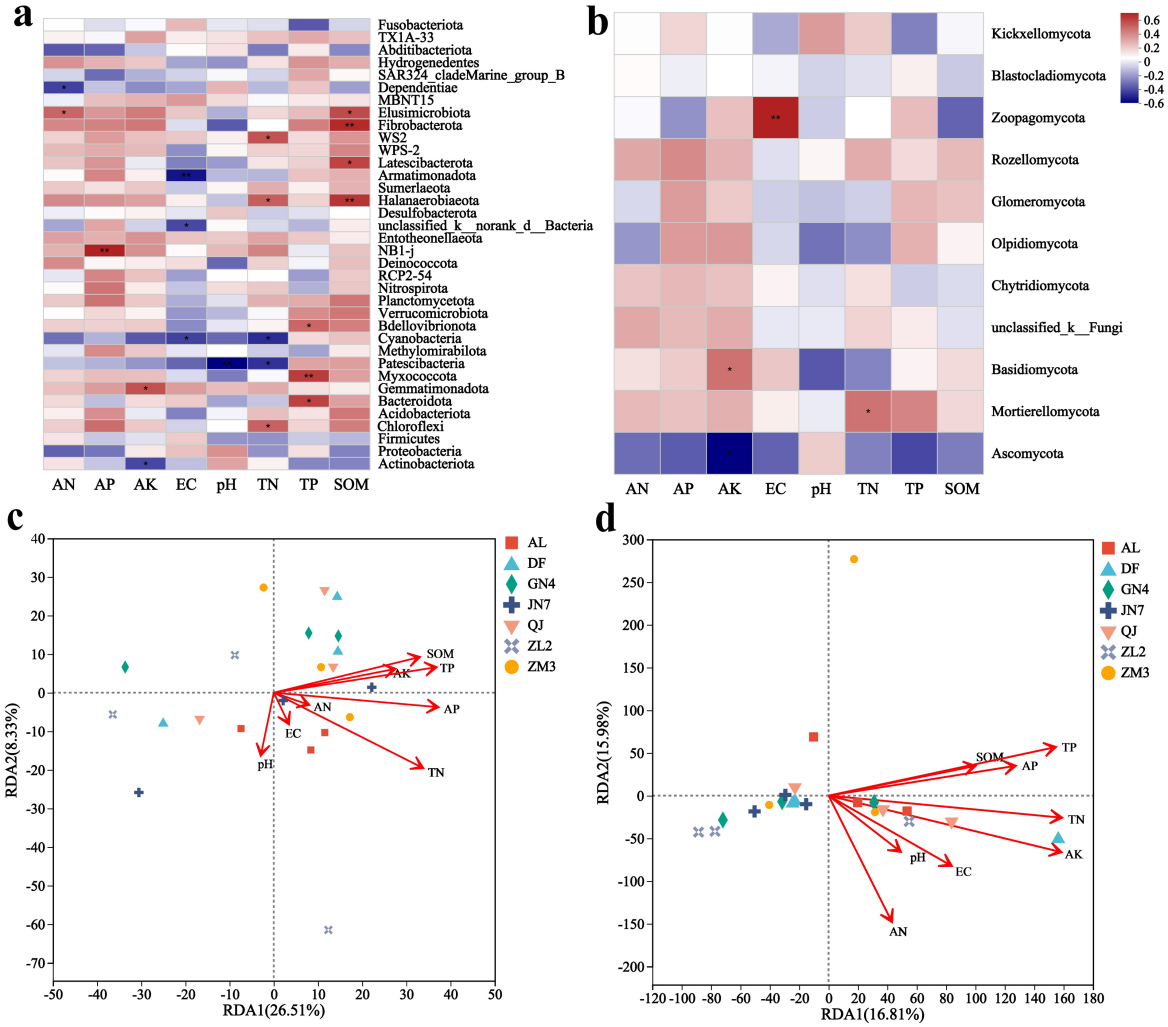
Spearman’s rank correlation coefficients (*R*) and statistical significance of the relationship between phylum abundance and soil factors at the bacterial **(a)** and fungal **(b)** levels. The f dbRDA analysis of bacterial **(c)** and fungal **(d)** communities. The *X*-axis and *Y*-axis represent environmental factors and microbial taxa, respectively. *R* values are displayed in different colors on the plot, while *p* values less than 0.05 are marked with *. The legend on the right shows color ranges for different *R* values. * *p* ≤ 0.05, ** *p* ≤ 0.01.

Multivariate analysis revealed significant interspecific variations in rhizosphere microbiomes among the alfalfa cultivars. PCoA based on Bray–Curtis distances revealed that the cultivar was a key determinant of bacterial community structure (PCoA2: 18.88%), whereas fungal assemblages exhibited different distributional patterns (PCoA1: 32.19%) (Figure 4, Table 2). For bacterial communities (Figure 5a; R = 0.3308, *p* = 0.002), PCoA1 and PCoA2 explained 27.01 and 18.88% of the observed variation, respectively. Bacterial communities across the seven cultivars clustered into two distinct groups, with samples from ZM3, GN4, DF, QJ, and AL showing close similarity, while ZL2 showed clear separation. The fungi (Figure 5b; *R* = −0.0234, *p* = 0.057) were more compactly clustered across the seven cultivars, and PCoA1 and PCoA2 together accounted for 45.51% of the fungal variation. This demonstrated that the fungal community structure was similar across the seven alfalfa cultivars. Furthermore, the PERMANOVA model attributed 55% of the variance in the bacterial community to the cultivar factor (Table 2, F.model = 2.83, *R*^2^ = 0.55, *p* = 0.001), indicating a strong cultivar-dependent effect under our experimental conditions. We postulate that this high explanatory power reflects the pronounced and systematic differences in root exudate profiles among the cultivars. Conversely, fungal diversity did not exhibit significant cultivar-dependent variation (F.model = 1.04, *R*^2^ = 0.31, *p* = 0.12).

**Table 2:** PERMANOVA of the effects of alfalfa cultivars on rhizosphere microbial communities.

Factor	Bacterial community			Fungal community		
	F	R^2^	*p*	F	R^2^	*p*
Cultivars	2.83	0.55	0.001	1.04	0.31	0.12

F represents F_model, R^2^ represents variation, and *p* represents the *p*-value.

### 3.5 Relationships between rhizosphere microbiome and soil factors

Soil physicochemical properties (pH, AN, AP, AK, TP, TN, and SOM) were analyzed using standard analytical methods (Table 3). All samples were collected from alfalfa cultivars grown in the same year and at the same geographic location to ensure comparability. Significant differences (*p* < 0.05) were observed only for soil pH among cultivars. However, the levels of AN, AP, and TN were comparable among ZM3, ZL2, DF, QJ, AL, and JN7 but were significantly higher in these cultivars than in GN4. TP levels in ZL2 and SOM levels in QJ were lower than those in the other cultivars. These differences likely reflected the cultivar-specific root exudate profiles and their associated rhizosphere microbial communities that modify nutrient cycling.

**Table 3:** Physicochemical properties of soil samples from different cultivars.

Samples	pH	AN (mg/kg)	AP (mg/kg)	AK (mg/kg)	TP (g/kg)	SOM (g/kg)	TN (g/kg)	EC (μs/cm)
M3	7.73 ± 0.08d	54.1 ± 19.6a	53.6 ± 6.61a	0.25 ± 0.04a	3.45 ± 0.13a	31.6 ± 1.03a	1.89 ± 0.34a	875. ± 11.6a
GN4	7.92 ± 0.01c	52.2 ± 12.9a	42.8 ± 9.53a	0.19 ± 0.04a	3.66 ± 0.13a	28.9 ± 9.57a	1.54 ± 0.38a	884. ± 4.50a
ZL2	8.00 ± 0.09bc	59.2 ± 14.1a	55.2 ± 4.36a	0.22 ± 0.08a	3.10 ± 0.26a	32.8 ± 6.84a	2.22 ± 0.14a	912. ± 67.3a
DF	8.08 ± 0.05ab	57.4 ± 6.41a	49.5 ± 4.41a	0.25 ± 0.14a	3.61 ± 0.28a	30.2 ± 2.43a	1.91 ± 0.26a	875. ± 4.59a
QJ	8.14 ± 0.01a	58.8 ± 14.6a	49.7 ± 7.95a	0.25 ± 0.06a	3.31 ± 0.02a	27.1 ± 1.19a	1.80 ± 0.07a	920. ± 49.8a
AL	8.1 ± 0.07ab	56.9 ± 17.6a	54.7 ± 7.15a	0.21 ± 0.06a	3.12 ± 0.40a	33.0 ± 6.88a	2.1 ± 0.45a	876. ± 1.06a
JN7	8.18 ± 0.03a	51.8 ± 6.41a	48.9 ± 4.56a	0.27 ± 0.09a	3.14 ± 0.29a	26.7 ± 1.84a	1.81 ± 0.03a	904. ± 4.24a

The data are presented as the mean ± standard errors (*n* = 3), with different superscript letters indicating significant differences (*p* < 0.05) among the seven cultivars. One-way analysis of variance was used to test for differences in soil chemical properties among different treatment groups. For multiple comparisons, Tukey’s Honest Significant Difference test was employed.

To examine the influence of soil parameters on microbial taxa, Spearman correlation analysis was performed between soil properties and microbial genera. Bacterial communities exhibited stronger correlations with soil physicochemical properties than did fungal communities (Figures 5a,b). Dominant *Medicago sativa* L. rhizosphere microbes showed differential responses to edaphic factors, with the influence of environmental factors being greater on bacteria than on fungi. Among them, SOM, TN, and TP displayed broad influence on bacterial communities, significantly affecting the abundance of *Elusimicrobia*, *Fibrobacterota*, *Latescibacterota*, *Halanaerobiaeota*, and *Myxococcota* (Figure 5a). However, only TN affected fungal communities. Fungal taxa, including *Zoopagomycota*, *Basidiomycota*, and *Mortierellomycota*, responded primarily to AK, pH, and TN, respectively (Figure 5b).

dbRDA analysis identified the distinct environmental drivers for bacterial and fungal communities. Bacterial community composition was strongly influenced by TN, AP, TP, and SOM (Figure 5c), whereas fungal communities responded mainly to TN, AK, and AP (Figure 5d). Notably, TP and SOM were positively correlated with the bacterial community of ZM3, while TN and AK strongly influenced the fungal profiles of AL. Although TP correlated with specific fungal OTUs (as indicated by Spearman’s correlation test), its independent contribution to overall fungal community structure appeared to be limited when other key factors were jointly considered.

### 3.6 Co-occurrence patterns among rhizosphere bacteria and fungi

**Figure 6:**
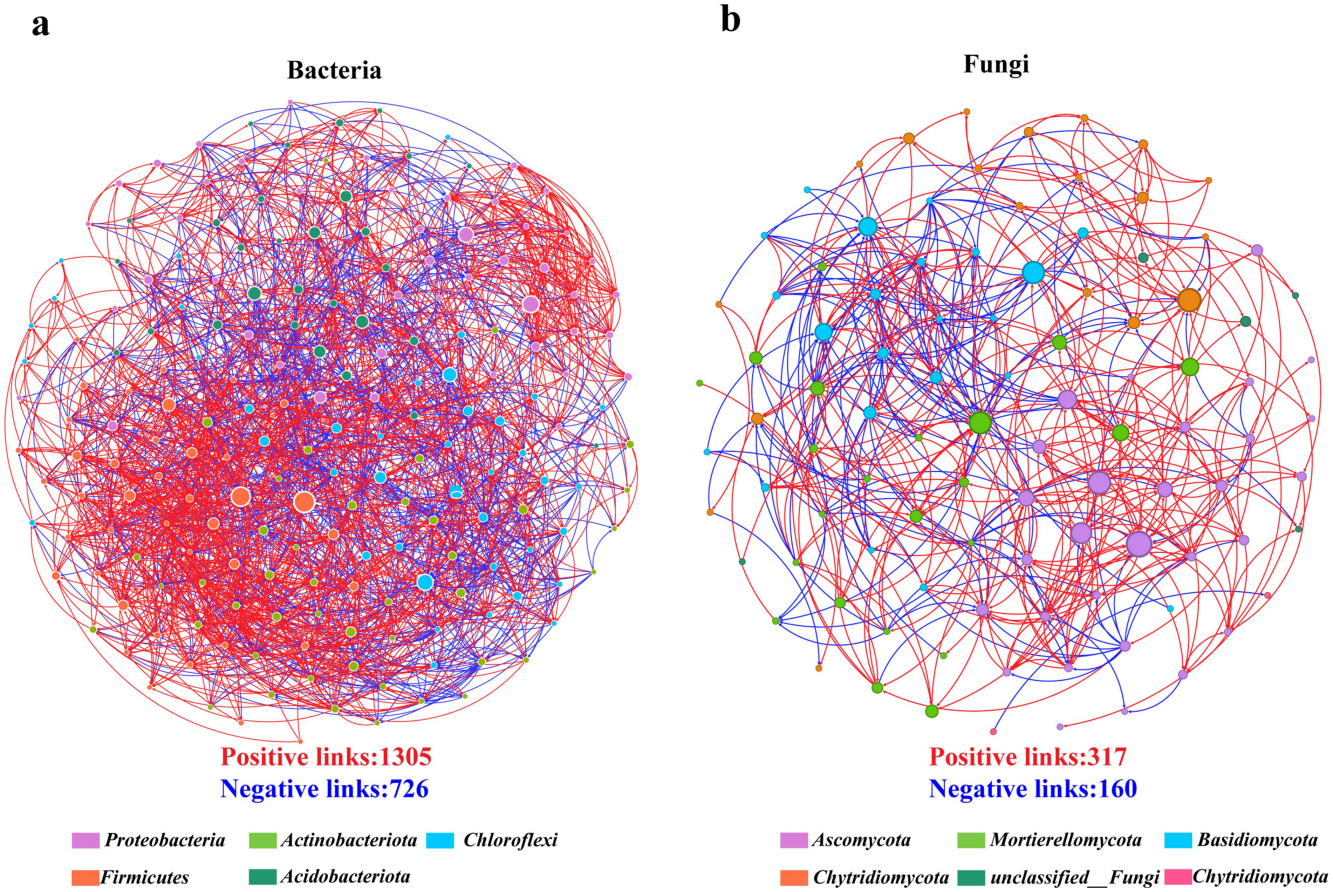
Network analysis of bacterial **(a)** and fungal **(b)** interactions in the rhizosphere soil. The size of the nodes is proportional to the number of links in each node. The lines between each pair of nodes represent positive (in red) and negative (in blue) interactions, with *p* < 0.05 and Spearman’s correlation coefficient over 0.5 or less than −0.5.

Correlation-based network analysis was conducted to characterize bacterial–fungal co-occurrence networks in the rhizospheres of the seven alfalfa cultivars. Network analysis revealed significant variations in the structure of the ecological network across different alfalfa cultivars. The number of positive correlations substantially outnumbered the number of negative interactions, revealing a big difference between bacterial and fungal communities. Notably, the bacterial community showed a higher level of complexity and modular structure, such that the interactions among bacterial OTUs in the soil were higher than those among fungal OTUs. The network topology metrics indicated that bacterial communities exhibited greater interaction complexity (link number: 2,031) than fungal communities (link number: 477) (Figure 6; Table 4). Furthermore, both bacterial (positive edges = 1,305) and fungal (positive edges = 317) networks exhibited a predominance of positive correlations.

**Table 4:** Correlation network analysis of microbial communities in the seven alfalfa cultivars.

Microbial group	Network properties	AL	GN4	ZL2	DF	QJ	ZM3	JN7
Bacteria	Node numbers	200	198	199	199	199	199	199
	Edge numbers	7,195	6,736	6,411	6,316	6,845	6,332	7,014
	Positive edges	3,717	3,383	3,437	3,294	3,577	3,176	3,706
	Negative edges	3,478	3,353	2,974	3,022	3,268	3,156	3,308
Fungi	Node numbers	98	99	99	100	99	98	98
	Edge numbers	2,697	1,633	2053	1,686	1,398	1,561	1733
	Positive edges	1,485	799	1,041	844	794	1,001	1,088
	Negative edges	1,212	834	1,012	842	604	560	645

Additionally, the microbial correlation network also varied significantly among the seven alfalfa cultivars. The cultivar AL displayed particularly intricate bacterial (nodes = 200, edges = 7,195) and fungal (nodes = 98, edges = 2,697) correlations compared to the other cultivars. After AL, the bacterial network complexity was the highest in QJ (nodes = 199, edges = 6,845), followed by GN4 (nodes = 198, edges = 6,736), ZL2 (nodes = 199, edges = 6,411), ZM3 (nodes = 199, edges = 6,332), and DF (nodes = 199, edges = 6,316). Meanwhile, the fungal network complexity followed the following order: AL > ZL2 > JN7 > DF > GN4 > ZM3 > > QJ. Across all cultivars, positive correlations remained consistently more frequent than negative ones. The microbial network of AL showed the highest connectivity, average degree, and modularity, suggesting that more frequent and complex interspecies interactions occurred within its rhizosphere. Ecological theories generally posit that such elevated network complexity may confer greater resilience to environmental disturbances, although this requires validation through dynamic or perturbation-based experiments in this specific model.

## 4 Discussion

Rhizosphere soil represents a dynamic microbial habitat that significantly contributes to plant nutrition and health. Evidence indicates that the microbial composition of the rhizosphere is shaped by multiple edaphic factors (water, pH, salts, moisture, and nutrition) (Liu et al., 2020). Moreover, differences in plant species and even genotypes within the same species can affect the rhizosphere microbiome (Khanna, 1993; Lemanceau et al., 1995). Despite this understanding, comparative analyses of rhizosphere microbiota across various alfalfa cultivars remain limited. Therefore, in this study, high-throughput sequencing was employed to profile the bacterial and fungal communities in the rhizospheres of seven distinct alfalfa cultivars at maturity. The findings of this study complement current knowledge on rhizosphere microbiome assembly mechanisms in alfalfa, a major leguminous forage crop, at the intraspecific level and offer a theoretical basis for future microbiome-based cultivar breeding. Overall, the study demonstrated that the rhizosphere microbiome of alfalfa is primarily composed of nine bacterial phyla, namely, Actinobacteriota, Proteobacteria, Firmicutes, Chloroflexi, Acidobacteriota, Bacteroidota, Gemmatimonadota, Myxococcota, and Cyanobacteria, as well as dominant fungal phyla such as Ascomycota, Mortierellomycota, Basidiomycota, and Chytridiomycota. Moreover, the most dominant bacteria and fungi found in the alfalfa rhizosphere were concentrated in the Actinobacteria, Proteobacteria, Ascomycota, and Mortierellomycota phyla. This composition is highly consistent with the core microbial groups detected in the rhizospheres of numerous plant species. Previously, Proteobacteria, Actinobacteria, and Acidobacteria were identified as the dominant bacteria in the alfalfa rhizosphere under drought conditions (Xing et al., 2023). Similarly, Lei et al. (2018) and Niu et al. (2017) found that Proteobacteria, Acidobacteriota, and Firmicutes were the dominant bacteria in the rhizosphere soil. Meanwhile, Osman et al. (2017) reported that the rhizosphere of rice (*Oryza sativa*) is dominated by Proteobacteria, Acidobacteria, Chloroflexi, Bacteroidetes, and Gemmatimonadetes. Additionally, Essel et al. (2019) demonstrated that Actinobacteria, Proteobacteria, Ascomycota, and Mucoromycota are the predominant microbial phyla in cultivated cereal soils. Comprehensive analyses have revealed that, despite variations in root-zone microbial composition across different plant species, Proteobacteria, Actinobacteria, and Ascomycota consistently emerge as dominant phyla across multiple systems, in close alignment with the findings of this study. The evidence indicates that these three microbial groups may represent the core dominant taxa widely present in diverse plant rhizosphere communities, exhibiting a certain degree of conservation and ecological universality.

Diversity metrics provide key insights for exploring ecological mechanisms. Lower diversity indices do not inherently signify inferior community quality, nor do they necessarily correlate with diminished stability or ecosystem health (Shade, 2017). In this study, although the rhizosphere microbial communities of the seven alfalfa varieties shared some overall structural similarity, key compositional differences remained. The ZM3 rhizosphere exhibited the highest bacterial diversity, whereas the AL rhizosphere showed the highest fungal diversity. Moreover, the QJ and JN7 rhizospheres stood out for their bacterial and fungal abundances, respectively. Furthermore, the JN7 rhizosphere was significantly enriched in Actinobacteria, Proteobacteria, and Ascomycota, while the QJ rhizosphere was significantly enriched in Actinobacteria, Proteobacteria, and Basidiomycota. These microbial groups are all closely linked to adaptive advantages in plants. First, the enriched Actinobacteria are recognized as key beneficial communities in arid soils, promoting stress tolerance. These microbes not only adapt to nutrient-poor and saline–alkali stresses but also produce antibiotics that suppress soil-borne pathogens, improve soil quality, and enhance plant growth (Wu et al., 2021). Second, the phylum Proteobacteria, the most abundant group in the rhizosphere, exhibits exceptional metabolic diversity and aids with nutrient acquisition. Many members of this phylum are highly efficient decomposers of organic matter and can serve as nitrogen fixers, directly promoting the cycling and uptake of key nutrients such as nitrogen and phosphorus by plants (Spain et al., 2009; Wang J. et al., 2020). Meanwhile, as primary saprophytic fungi, ascomycetes and basidiomycetes play indispensable roles in decomposing recalcitrant organic matter (Sansupa et al., 2022). Thus, based on the findings of this study, we infer that the JN7 and QJ cultivars may shape rhizosphere microenvironments that are capable of selectively enriching these beneficial microorganisms through their unique root exudates (e.g., specific organic acids and amino acids) and root morphology. These tailored microbial communities could theoretically enhance the stress resistance and nutrient mobilization efficiency of the host plant, thereby improving cultivation potential in nutrient-poor soils. This crucial microbiological evidence supports the value of JN7 and QJ as useful cultivars for alfalfa breeding. Subsequent studies should focus on directly isolating and validating specific microbial strains with growth-promoting and stress-resistant functions from the rhizospheres of these two cultivars.

The rhizosphere serves as a hub for plant–microbe interactions that facilitate nutrient and water uptake by plants (Castellano-Hinojosa et al., 2021). As such, the functional role of rhizosphere soil microbial communities is gaining increasing attention. Functional prediction tools such as PICRUSt and FUNGuild serve as effective hypothesis generators. Through functional analysis in this study, AAM and EPC emerged as the most important potential functional traits of alfalfa rhizosphere bacteria, consistent with the pivotal role of Proteobacteria and Actinobacteria in facilitating metabolism-related functions (Yan et al., 2021). Soil fungi play essential ecological roles and contribute to processes such as nutrient cycling, pest suppression, and plant vegetation dynamics (Devi et al., 2020). In this study, the most important potential functions of fungi were found to be Animal Pathogen–Endophyte–Lichen Parasite–Plant Pathogen–Wood Saprotroph and Plant Pathogen. According to Toju et al. (2016), diverse groups of fungi, including mycorrhizal, endophytic, saprotrophic, and pathogenic fungi, can establish modular networks in soil environments, characterized by cooperative, inhibitory, or competitive interactions. Our findings align with this hypothesis. Nevertheless, the microbial functional predictions generated by PICRUSt2 and FUNGuild are computational inferences, as amplicon data (particularly OTUs) typically lack sufficient resolution to distinguish lifestyle from function itself. Therefore, these results reflect potential functionality rather than real-time functions and require further validation through follow-up experiments. Therefore, the functional prediction data obtained in this study warrant further confirmation through direct methods such as metagenomics, metatranscriptomics, or biochemical assays.

Soil factors share a close association with the microbial composition and distribution in the rhizosphere. Local soil conditions such as pH, moisture, temperature, and nutrient availability directly modulate microbial viability (Che et al., 2019; Zhang et al., 2019; Lopes and Schachtman, 2023). Investigations of seven key soil physicochemical parameters in this study revealed that bacterial communities were the most affected by TP, TN, and SOM, whereas TP, TN, and AP had the most significant influence on fungal communities. Moreover, TP emerged as the predominant driver of microbial community structure. Interestingly, notable variations in soil parameters were observed across the seven alfalfa cultivars despite uniform climatic conditions and cultivation practices. For example, the pH of ZM3 was significantly lower than that of JN7, although its microbial diversity was higher. Given that ZM3 is a typical salt-tolerant alfalfa variety, we hypothesize that this phenomenon may be closely related to its salt tolerance. On the one hand, the root exudates of ZM3 may contain higher levels of organic acids, which could react with carbonates in the rhizosphere. These carbonates could undergo decomposition in neutral and alkaline soils, thereby lowering the pH. On the other hand, ZM3 may simultaneously recruit more salt-tolerant microorganisms that reduce soil pH by secreting organic acids. Concurrently, plant roots and associated microorganisms may alter rhizosphere pH through redox-coupling reactions (Philippe Hinsinger et al., 2003; Fu et al., 2023). Notably, evidence indicates that soil pH is inversely associated with bacterial and fungal diversity (Zhang et al., 2019b). Our results are consistent with previous findings, demonstrating that pH can serve as a major predictor of microbial diversity. Specifically, we observed variations in dominant microbial taxa across different cultivars, indicating that microbial communities actively modify the soil TP, AP, TN, and AK contents, with the continuous feedback loop between soil chemistry and the microbiota driving ecosystem dynamics. Nevertheless, it is important to emphasize that no non-rhizosphere soil was tested as a control in this study, and thus, whether microbial community alterations stem from the specific selection of alfalfa varieties or the residual effects of initial soil heterogeneity remains to be clarified. Future research should incorporate non-rhizosphere soil controls to quantify variety effects more precisely. Additionally, the statistical power of this study is limited by its sample size (*n* = 3). Increasing the number of biological replicates in future studies could help reveal more granular cultivar-specific differences.

Identifying and characterizing the interactions among soil microorganisms is critical for understanding microbial diversity and functional dynamics. Network analysis is a powerful tool for exploring these interactions (Shi et al., 2016). In this study, we conducted Spearman’s correlation analysis and combined it with random matrix theory to examine rhizosphere microbial networks across seven alfalfa cultivars. Our results showed that while the cultivars shared similar microbial network membership at the phylogenetic level, their network structures differed markedly, indicating cultivar-specific organizational patterns. Analysis of network edge counts indicated that bacterial networks exhibited more complex and denser interaction patterns than fungal networks, suggesting a more tightly interconnected bacterial community structure. Among all cultivars, AL exhibited the highest network complexity for both bacteria and fungi, followed by JN7, indicating higher ecosystem stability. Moreover, despite similar microbial community composition among the remaining cultivars, their network architectures showed substantial differences, suggesting that certain low-abundance or non-dominant genera may exert some ecological influence. Additionally, both bacterial and fungal networks exhibited more positive correlations than negative correlations, indicating that mutualistic relationships dominate over competitive interactions in the microbial community. This finding aligns with previous findings by Rybakova et al. (2017), who also observed that rhizosphere microbial communities are primarily characterized by co-occurrence rather than competitive exclusion. Notably, the majority of dominant taxa coexisted stably with each other and with specific low-abundance species. This study provides compelling evidence for cultivar-dependent assembly of alfalfa rhizosphere microbial communities and highlights the potential relevance for future alfalfa breeding strategies.

## 5 Conclusion

The results of this study revealed that alfalfa (*Medicago sativa* L.) cultivars significantly influence rhizosphere microbial communities, including their diversity, structure, predicted function, responses to soil factors, and co-occurrence networks. The core microbial phyla across cultivars were Actinobacteria, Proteobacteria, and Ascomycota. Functional predictions indicated that AAM and EPC were the most important bacterial traits, while fungal communities were dominated by functions associated with Animal Pathogen–Endophyte–Lichen Parasite–Plant Pathogen–Wood Saprotroph and Plant Pathogen. However, as these predictions were based on computational inference, they require further validation. Moreover, we found that edaphic factors differentially regulate microbial distribution. SOM, TN, and TP significantly influenced bacterial communities, while TN and AP primarily shaped fungal communities, with TN emerging as the predominant regulator of overall rhizosphere microbiota. Furthermore, network analyses further revealed stronger interconnectivity among bacterial communities compared with fungal assemblages in alfalfa cultivars, with cooperative interactions predominating over competitive relationships. Although these networks could aid in identifying interactions among microorganisms, they remain theoretical and require experimental validation. In summary, this study provides new evidence for substantial functional and structural differences in rhizosphere microbiomes among cultivars of the same species. They highlight the importance of plant genotype–microbiome interactions and offer a theoretical basis for developing molecular marker-assisted breeding strategies aimed at enhancing the plant’s recruitment capacity for beneficial microbes. Such strategies could hold considerable potential for advancing sustainable agricultural practices.

## Data Availability

The sequencing data analyzed in the current study are available through the NCBI Sequence Read Archive under BioProject PRJNA1047311.

## Footnotes

1
https://github.com/OpenGene/fastp

2
https://cloud.majorbio.com
