# Supplementary material for: Variation in the structure of microbial communities associated with different alfalfa (Medicago sativa L.) cultivars
Source: Front Microbiol. 2026 Jan 6;16:1676231. doi: 10.3389/fmicb.2025.1676231 (PMC12815815; doi:10.3389/fmicb.2025.1676231)
Supplement: Supplementary file 1 [file Data_Sheet_1.docx]

Supplementary Material

# Supplementary Figures


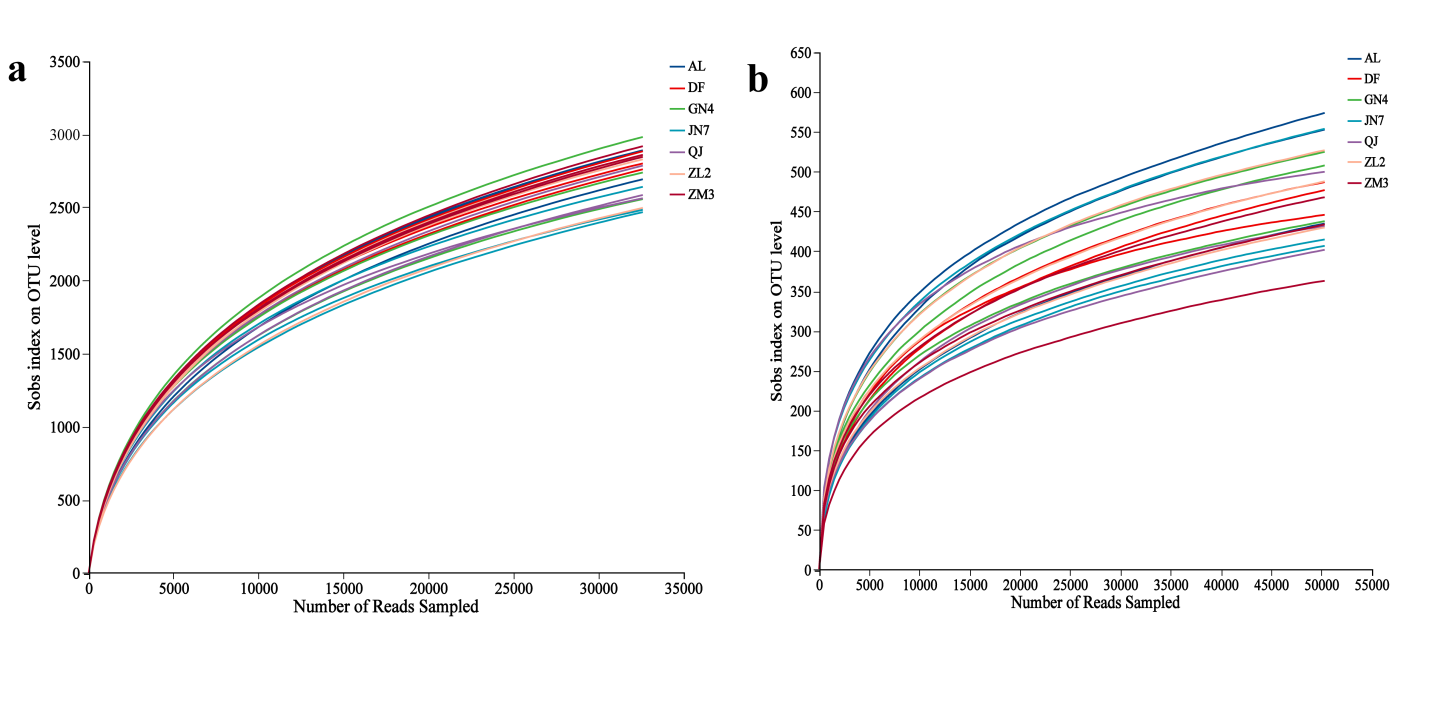


**Supplementary Figure 1.** Rarefaction curve of rhizosphere soil bacteria (a) and fungi (b) communities in different alfalfa cultivars

**
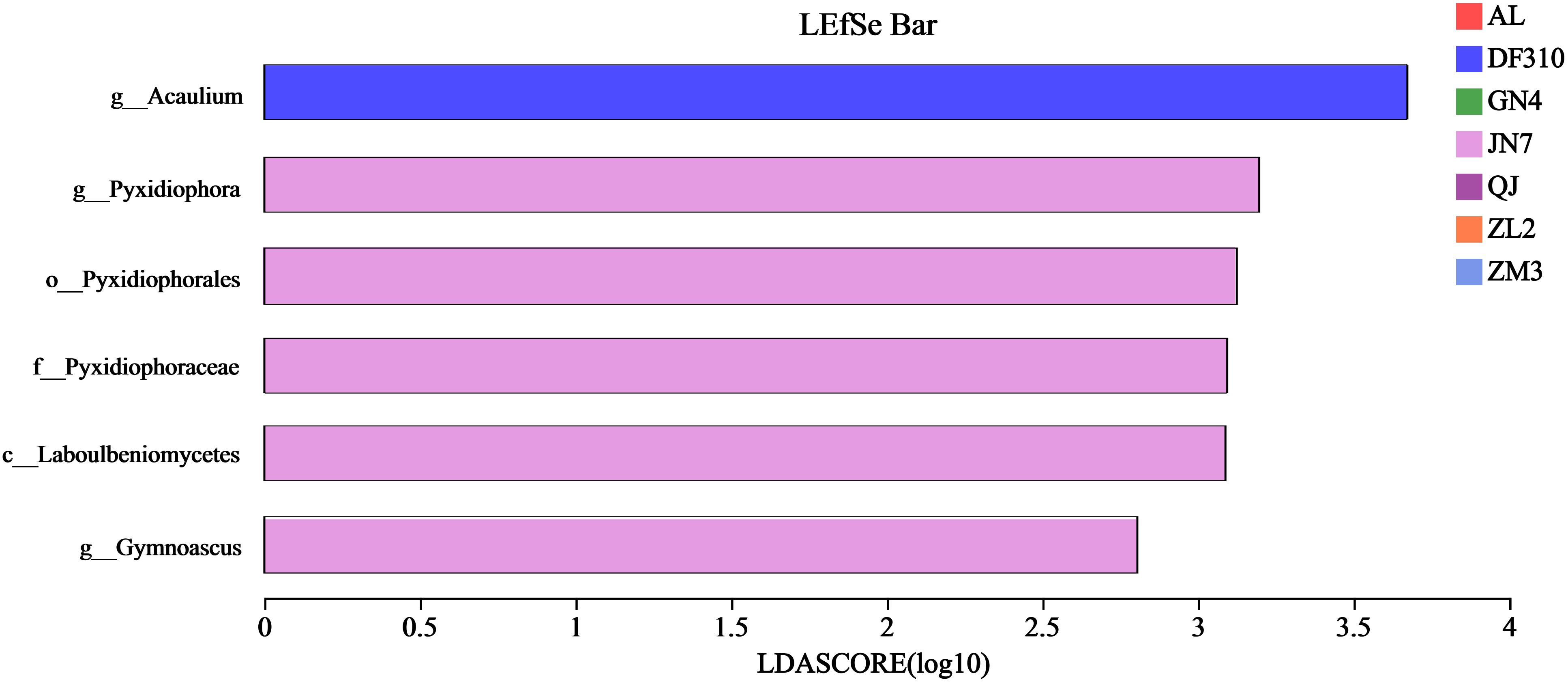
**

**Supplementary Figure 2.** Multilevel differential species analysis of rhizosphere soilfungi (b) among alfalfa cultivars


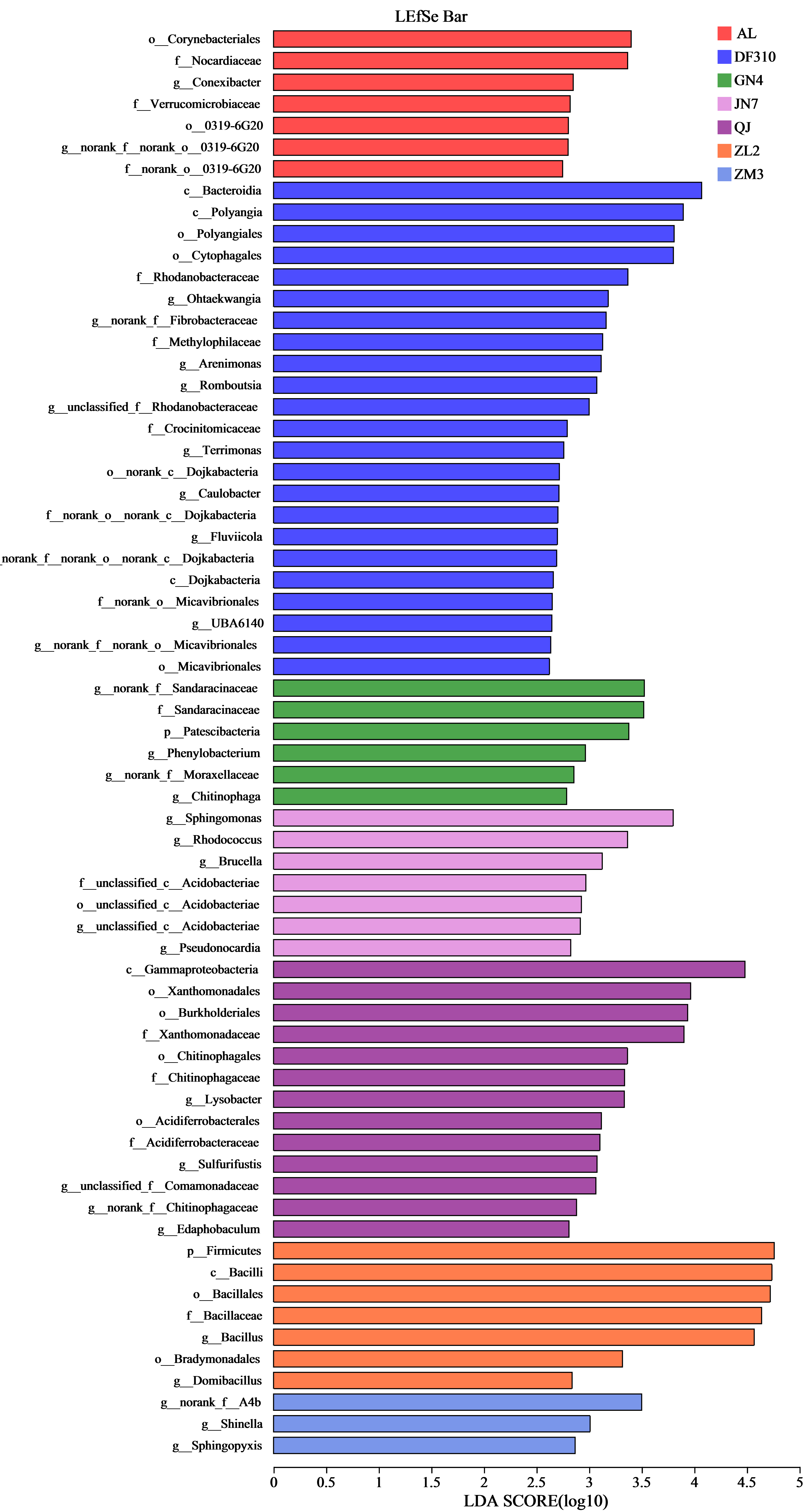


**Supplementary Figure 3.** Multilevel differential species analysis of rhizosphere soil bacteria among alfalfa cultivars
